# Risk Factors and Time to Clinical Symptoms of Multiple Sclerosis Among Patients With Radiologically Isolated Syndrome

**DOI:** 10.1001/jamanetworkopen.2021.28271

**Published:** 2021-10-11

**Authors:** Christine Lebrun-Frénay, Fabien Rollot, Lydiane Mondot, Helene Zephir, Celine Louapre, Emmanuelle Le Page, Françoise Durand-Dubief, Pierre Labauge, Caroline Bensa, Eric Thouvenot, David Laplaud, Jerome de Seze, Jonathan Ciron, Bertrand Bourre, Philippe Cabre, Olivier Casez, Aurélie Ruet, Guillaume Mathey, Eric Berger, Thibault Moreau, Abdulatif Al Khedr, Nathalie Derache, Pierre Clavelou, Anne-Marie Guennoc, Alain Créange, Jean-Philippe Neau, Ayman Tourbah, Jean-Philippe Camdessanché, Adil Maarouf, Celine Callier, Patrick Vermersch, Orhun Kantarci, Aksel Siva, Christina Azevedo, Naila Makhani, Mikael Cohen, Daniel Pelletier, Darin Okuda, Sandra Vukusic

**Affiliations:** 1 Centre de Resssource et Competence Sclérose En Plaques Nice, Unité Recherche Clinique Cote d'Azur Unité de Recherche sur le Syndrome Radiologique Isolé, Université Nice Côte d’Azur, Neurologie Centre Hospitalier Universitaire Pasteur 2, Nice, France; 2 Centre des Neurosciences de Lyon, Observatoire Français de la Sclérose en Plaques, Institut National de la Santé et de la Recherche Médicale 1028 et Centre National de Recherche Scientifique Unité Mixte de Recherche 5292, Lyon, France Université Claude Bernard Lyon 1, Lyon, France; 3 European Database for Multiple Sclerosis Foundation, Lyon, France; 4 Université Claude Bernard Lyon 1, Faculté de Médecine Lyon Est, Lyon, France; 5 Université de Lille, Inserm Unité Mixte de Recherche-S 1172 LilNcog, Centre Hospitalier Universitaire Lille, Fédération Hospitalo-Universitaire Precise, Lille, France; 6 Sorbonne University, Department of Neurology, Assistance Publique des Hôpitaux de Paris, Pitié-Salpêtrière Hospital, Paris, France; 7 Centre Hospitalier Universitaire Pontchaillou, Centre d'Investigation Clinique 1414 Institut National de la Santé et de la Recherche Médicale, Rennes, France; 8 Service de Neurologie, Sclérose en Plaques, Pathologies de la Myéline et Neuro-Inflammation, Hôpital Neurologique Pierre Wertheimer, Hospices Civils de Lyon, Lyon, France; 9 Centre de Ressources et Competences Sclerose En Plaques, Centre Hospitalier Universitaire de Montpellier, Montpellier, France; 10 University of Montpellier, Montpellier, France; 11 Department of Neurology, Fondation Rothschild, Paris, France; 12 Department of Neurology, Centre Hospitalier Universitaire de Nîmes, Nîmes, France; Institut de Génomique Fonctionnelle, Université de Montpellier, Centre National de Recherche Scientifique, Institut National de la Santé et de la Recherche Médicale, Montpellier, France; 13 Service de Neurologie, Centre d'Investigation Clinique 015 Institut National de la Santé et de la Recherche Médicale, Nantes, France; Institut National de la Santé et de la Recherche Médicale 1064, Nantes, France; 14 Department Clinical Investigation Center, Department of Neurology, Centre Hospitalier Universitaire de Strasbourg, Institut National de la Santé et de la Recherche Médicale 1434, Strasbourg, France; 15 Department of Neurology, Centre de Resssource et Competence Sclérose En Plaques, Centre Hospitalier Universitaire de Toulouse; Institut Toulousain des Maladies Infectieuses et Inflammatoires (Infinity), Institut National de la Santé et de la Recherche Médicale Unité Mixte de Recherche1291, Centre National de Recherche Scientifique Unité Mixte de Recherche 5051, Université Toulouse III Toulouse, France; 16 Department of Neurology, Centre Hospitalier Universitaire de Rouen, Rouen, France; 17 Department of Neurology, Centre Hospitalier Universitaire de la Martinique, Fort-de-France, France; 18 Department of Neurology, Centre Hospitalier Universitaire Grenoble Alpes, Grenoble, France; 19 Centre de Resssource et Competence Sclérose En Plaques, Neurology Department, Centre Hospitalier Universitaire of Bordeaux, Bordeaux, France; Université Bordeaux, Institut National de la Santé et de la Recherche Médicale, Neurocentre Magendie, U1215, Bordeaux, France; 20 Department of Neurology, Nancy University Hospital, Nancy, France; Université de Lorraine, Equipe Avenir 4360 Adaptation, Mesure et Evaluation en Sante Approches Interdisciplinaires, Vandoeuvre-Lès-Nancy, Nancy, France; 21 Department of Neurology, Centre Hospitalier Universitaire de Besançon, Besançon, France; 22 Department of Neurology, Centre Hospitalier Universitaire de Dijon, EA4184, Dijon, France; 23 Department of Neurology, Centre Hospitalier Universitaire d’Amiens, Amiens, France; 24 Department of Neurology, Centre Hospitalier Universitaire de Caen Normandie, Caen, France; 25 Department of Neurology, Neuro-Dol, Centre Hospitalier Universitaire Clermont-Ferrand, Université Clermont Auvergne, Institut National de la Santé et de la Recherche Médicale U1107, Clermont-Ferrand, France; 26 Department of Neurology, Centre Hospitalier Universitaire de Tours, Hôpital Bretonneau, Centre de Resssource et Competence Sclérose En Plaques, Tours, France; 27 Department of Neurology, Assistance Publique des Hôpitaux de Paris, Hôpital Henri Mondor, Université Paris Est, Créteil, France; 28 Department of Neurology, Centre Hospitalier Universitaire de Poitiers, Poitiers, France; 29 Department of Neurology, Hôpital Raymond Poincaré, Garches, Unité de Formation de Recherche Simone Veil, Institut National de la Santé et de la Recherche Médicale U1195, Assistance Publique Hopitaux de Paris, Université Paris Saclay, France; 30 Department of Neurology, Centre Hospitalier Universitaire de Saint-Étienne, Hôpital Nord, Saint-Étienne, France; 31 Department of Neurology, Centre Hospitalier Universitaire Timone, Marseille, France; 32 Mayo Clinic, Rochester, Minnesota; 33 Department of Neurology, Istanbul University Cerrahpasa School of Medicine, Turkey; 34 University of Southern California, Los Angeles; 35 Departments of Pediatrics and Neurology, Yale School of Medicine, New Haven, Connecticut; 36 University of Texas Southwestern Medical Center, Dallas

## Abstract

**Importance:**

Younger age, oligoclonal bands, and infratentorial and spinal cord lesions are factors associated with an increased 10-year risk of clinical conversion from radiologically isolated syndrome (RIS) to multiple sclerosis (MS). Whether disease-modifying therapy is beneficial for individuals with RIS is currently unknown.

**Objectives:**

To evaluate the 2-year risk of a clinical event (onset of clinical symptoms of MS) prospectively, identify factors associated with developing an early clinical event, and simulate the sample size needed for a phase III clinical trial of individuals with RIS meeting 2009 RIS criteria.

**Design, Setting, and Participants:**

This cohort study used data on prospectively followed-up individuals with RIS identified at 1 of 26 tertiary centers for MS care in France that collect data for the Observatoire Français de la Sclérose en Plaques database. Participants were aged 10 to 80 years with 2 or more magnetic resonance imaging (MRI) scans after study entry and an index scan after 2000. All diagnoses were validated by an expert group, whose review included a double centralized MRI reading. Data were analyzed from July 2020 to January 2021.

**Exposure:**

Diagnosis of RIS.

**Main Outcomes and Measures:**

Risk of clinical event and associated covariates at index scan were analyzed among all individuals with RIS. Time to the first clinical event was compared by covariates, and sample size estimates were modeled based on identified risk factors.

**Results:**

Among 372 individuals with RIS (mean [SD] age at index MRI scan, 38.6 [12.1] years), 354 individuals were included in the analysis (264 [74.6%] women). A clinical event was identified among 49 patients (13.8%) within 2 years, which was associated with an estimated risk of conversion of 19.2% (95% CI, 14.1%-24.0%). In multivariate analysis, age younger than 37 years (hazard ratio [HR], 4.04 [95% CI, 2.00-8.15]; *P* < .001), spinal cord lesions (HR, 5.11 [95% CI, 1.99-13.13]; *P* = .001), and gadolinium-enhancing lesions on index scan (HR, 2.09 [95% CI, 1.13-3.87]; *P* = .02) were independently associated with an increased risk of conversion to MS. Having 2 factors at the time of the index MRI scan was associated with a risk of 27.9% (95% CI, 13.5%-39.9%) of a seminal event within 2 years, increasing to 90.9% (95% CI, 41.1%-98.6%) for individuals with all 3 factors (3 risk factors vs none: HR, 23.34 [95% CI, 9.08-59.96]; *P* < .001). Overall, with 80% power to detect an effect size of 60% within 24 months, a total of 160 individuals with RIS were needed assuming an event rate of 20%.

**Conclusions and Relevance:**

This study found that age younger than age 37 years, spinal cord involvement, and gadolinium-enhancing lesions on index MRI scan were associated with earlier clinical disease and relevant to the number of enrolled patients needed to detect a potential treatment effect.

## Key Points

**Question:**

Are there clinical or demographic factors associated with time to clinical symptoms of multiple sclerosis among patients with radiologically isolated syndrome?

**Findings:**

In this cohort study of 372 individuals with radiologically isolated syndrome, young age, the presence of spinal cord lesions, and gadolinium-enhancing lesions on the index magnetic resonance imaging scan were associated with increased risk of onset of clinical symptoms of multiple sclerosis.

**Meaning:**

These findings identify 3 risk factors associated with multiple sclerosis at the preclinical stage of central nervous system demyelinating disease defined by radiologically isolated syndrome.

## Teaser

This cohort study investigates risk factors associated with time to a multiple sclerosis clinical event among patients with radiologically isolated syndrome.

## Introduction

Since the 1980s, preclinical multiple sclerosis (MS) was identified as brain magnetic resonance imaging (MRI) findings consistent with central nervous system (CNS) demyelination incidentally discovered among individuals without symptoms.^1,2,3,4,5^ In 2009, criteria for radiologically isolated syndrome (RIS) were defined to enhance the characterization of patients.^3^ Demographics of individuals with RIS later evolved to include relapsing or progressive subtypes of MS mirroring the different clinical forms of MS^6,7,8,9^ among adults and children.^10^

An analysis of sizeable multicenter retrospective data collected by the RIS Consortium (RISC) found that younger age, male sex, and the presence of spinal cord lesions were associated with 5-year-risk of conversion to a first clinical demyelinating event, suggesting the importance of intramedullary lesions on clinical evolution.^11,12^ After 10 years, age and spinal cord lesions were still associated with increased risk, as were oligoclonal bands (OCBs) in cerebrospinal fluid (CSF) and infratentorial lesions on the index scan^13^ and occurrences of gadolinium-enhancing lesions on follow-up MRIs.

Despite this homology with MS, the association of early treatment with decreased conversion to MS is unknown. Two multicenter, randomized, double-blinded clinical trials evaluated the efficacy of dimethyl fumarate (NCT02739542)^14^ and teriflunomide (NCT03122652)^15^ vs placebo in delaying time to the first clinical event. In this study, we evaluated the 2-year risk of a first clinical event suggestive of MS and estimated sample sizes needed for 24-month prospective clinical trials based on the primary outcome of a first clinical event and critical risk factors that may enrich cohorts studied.

## Methods

### Ethics Statement

The French MS Registry (Observatoire Français de la Sclérose En Plaques [OFSEP]) was approved by the French regulatory authorities (Commission Nationale Informatique et Libertés) and ethics committee (Comité de Protection des Personnes). Patients enrolled in OFSEP provide written consent for their participation.^16^ This cohort study used OFSEP data and is compliant with French regulatory and General Data Protection Regulation requirements, including informed consent. This study followed the Strengthening the Reporting of Observational Studies in Epidemiology (STROBE) reporting guideline.

### Study Design and Participants

We conducted a French multicenter study with 26 tertiary centers for MS care collecting data for the OFSEP registry.^16^ Individuals fulfilling 2009 criteria for RIS^3^ were enrolled prospectively (eTable 1 in Supplement 1). Diagnoses of RIS were validated by an expert group (C.L.F., L.M., O.K., A.S., C.A., D.P., and D.O.), including performance of a centralized MRI reading. After the adjudication of clinical and radiological criteria, the diagnosis of RIS was validated.

Inclusion criteria were age 10 to 80 years, adjudicated diagnosis of RIS, availability of 2 or more MRI scans after study entry, and index MRI scan after 2000. Exclusion criteria were history of neurological symptoms suggestive of CNS demyelination, incomplete MRI sequences, and absence of follow-up. All MRI scans fulfilling 2005 MS dissemination in space criteria^17^ were centralized for documentation.

Demographics, family history of MS, detailed historical and current clinical data, comprehensive neurological examination results, index brain and spinal cord MRI findings, and CSF analysis were collected at study entry. Data were recorded using standardized case report forms provided to neurologists to record the onset and characteristics of any relapsing or progressive neurologic symptoms and the presence and location of new MRI lesions during follow-up. Data were digitized using the European Database for Multiple Sclerosis (EDMUS) software version 5.7.1 (EDMUS).^18^

### Procedures

At baseline and at 1 or more follow-up visits for each patient, the individual’s characteristics and neurological examinations were collected. Variables recorded included demographic characteristics (eg, age at the time of RIS diagnosis and sex), clinical data (ie, MS family history, reason for index MRI scan, and use of disease-modifying treatments [DMTs] during RIS phase), and imaging data (ie, number of lesions ≥3mm^2^ on T2-weighted axial sequences and presence of gadolinium-enhancing lesions in the brain and spinal cord).

Positive CSF was defined as an IgG index of 0.7 or greater or as the presence of 2 or more oligoclonal bands in the CSF that were not present in a corresponding serum sample. A seminal clinical event was defined as developing (1) a subacute neurological episode lasting more than 24 hours in the absence of fever or acute illness or (2) the onset of a clinical symptom with temporal profile revealing at least a 12-month progression of neurological deficits.

### Neuroimaging Studies

All individuals had an initial brain MRI scan that revealed incidental anomalies suggestive of a demyelinating disease and fulfilled 2009 criteria for RIS.^3^ Follow-up brain and spinal cord MRI scans were acquired following the OFSEP MRI protocol^19^ on 1.5 or 3 Tesla machines. Abnormalities within the brain or spinal cord were initially identified by a local neurologist or radiologist (C.L.F., H.Z., C.L., E.L.P., F.DD., P.L., C.B., E.T., D.L., J.D.S., J.C., B.B., P.C., O.C., A.R., G.M., E.B., T.M., A.A.K., N.D., P.C., A.M.G., A.C., J.P.N., A.T., J.P.C., A.M., P.V., M.C., and S.V.) and then centralized and independently reviewed by^2^ MS specialists (C.L.F. and L.M.) to ensure MRI criteria for RIS were met.^3^

### Statistical Analysis

When appropriate, means with SDs were calculated to summarize demographic, clinical, and radiological data. The association of each covariate at index scan with time to the first clinical symptom was quantified by hazard ratios (HRs) with 95% CIs using standard survival analysis methods in unadjusted and adjusted Cox proportional hazards models. Proportionality assumption was tested by a global test based on Schoenfeld residuals analysis^20^ and the graphical and numerical methods of Lin et al.^21^ First, covariates with a *P* value < .20 in univariate analyzes were included in the multivariate modeling strategy. A backward and stepwise variable selection procedure by *P* value and Akaike information criteria was used to select final multivariate models. Time to first clinical event was estimated with Kaplan-Meier curves. A DMT forced in the final model was considered a time-varying covariate to limit the bias associated with treatment indication. All analyses were censored at 2 years from the index scan.

We estimated the number of individuals needed using a Cox regression to achieve a power of 80%, assuming a 5% type 1 error rate. We simulated scenarios with several assumptions on the event rate based on our results on actual data (ie, event rates of 15%, 20%, 30%, and 90%) and the effect size of a binary covariate (such as the treatment effect) ranging from a decrease of 40% to a decrease of 70%.

Statistical analyses were performed using SAS statistical software version 9.4 (SAS Institute) and R statistical software version 3.5.0 (R Project for Statistical Computing). PASS software version 2008 (NCSS) was used to estimate the number of individuals needed. A 2-sided *P* value < .05 was considered statistically significant. Data were analyzed from July 2020 to January 2021.

## Results

### Clinical Characteristics

Among 372 individuals fulfilling 2009 RIS criteria,^3^ the mean (SD) age at RIS diagnosis was 38.6 (12.1) years and the mean (SD) clinical follow-up time overall was 3.8 (3.6) years. Among all patients, 48 individuals (12.9%) had a familial history of MS and 15 individuals (4.2%) were diagnosed with RIS before age 18 years. We excluded 18 individuals because they had no follow-up after index scan, and 57 patients (16.1%) from the remaining population were considered lost to follow-up (ie, patients without conversion and not followed 2 years and thus censored at the last clinical visit). However, when comparing patients lost to follow-up with others, there were no significant differences in terms of patient characteristics, which made it possible to limit the bias associated with the informative censoring. Among 354 individuals in the final cohort, there were 264 (74.6%) women.

Among 38 individuals (1.1%), MRI was performed because of a familial history of MS. We performed CSF analysis among 202 individuals, and results were abnormal (ie, OCBs and or high index) in 88 of 202 available results (43.6%).

A DMT was prescribed before the first clinical episode for 61 patients (17.2%), corresponding to 75 DMTs, using 8 different European Medicines Agency–approved treatments for relapsing forms of MS (eTable 2 in Supplement 1). Treatment decisions were made on an individual basis at the discretion of the referring physician. Among all DMTs, 26 treatments (34.7%) were first-line injectable medications (ie, interferon beta or glatiramer acetate), 22 treatments (29.3%) were first-line oral drugs (ie, teriflunomide or dimethyl fumarate), 9 treatments (12.0%) were second line immunosuppressors (ie, fingolimod or natalizumab), and 5 treatments (6.7%) were other nonapproved drugs (ie, azathioprine, mycophenolate mofetil, methotrexate, mitoxantrone, or rituximab). The mean (SD; range) cumulative treatment duration was 10.07 (6.88; 0.03-24.79) months.

### Clinical Event

A first clinical event suggestive of CNS demyelination at 2 years was identified among 49 patients (13.8%). Among these individuals, there were 35 (71.4%) women, 38 individuals (77.6%) aged younger than 37 years, and 9 individuals (18.4%) who were polysymptomatic. The first clinical event was myelitis for 29 individuals (46.9%), optic neuritis for 9 individuals (18.4%), long tracts for 8 individuals (16.3%), brainstem syndrome for 5 individuals (10.2%), and unknown for 3 individuals (6.1%). The mean (SD; range) Expanded Disability Status Scale (EDSS) score at the clinical event was 1.71 (1.94; 0-8.00); data were missing for 16 (32.7%) individuals. Among all 49 individuals with events, 23 individuals (46.9%) had been treated with at least 1 DMT and 5 individuals (10.2%) fulfilled the criteria for primary progressive MS.

### Reasons for MRI Index Scan

Reasons for the brain MRI index scan were diverse, with headache (101 individuals [28.5%]) being the most common, followed by ears, nose, and throat concerns (53 individuals [15.0%]), witness in clinical trial participation (39 individuals [11.0%]), ophthalmological disease not related to MS (ie, macular edema, ocular trauma, or infection; 18 individuals [5.1%]), and cranial trauma (16 individuals [4.5%]). Headache was not associated with a clinical event at 2 years (HR = 0.64 [95% CI, 0.32-1.28]; *P* = .20) (eFigure 1 in Supplement 1).

### 2-Year Risk of Clinical Event Suggestive of MS

**Figure 1.**
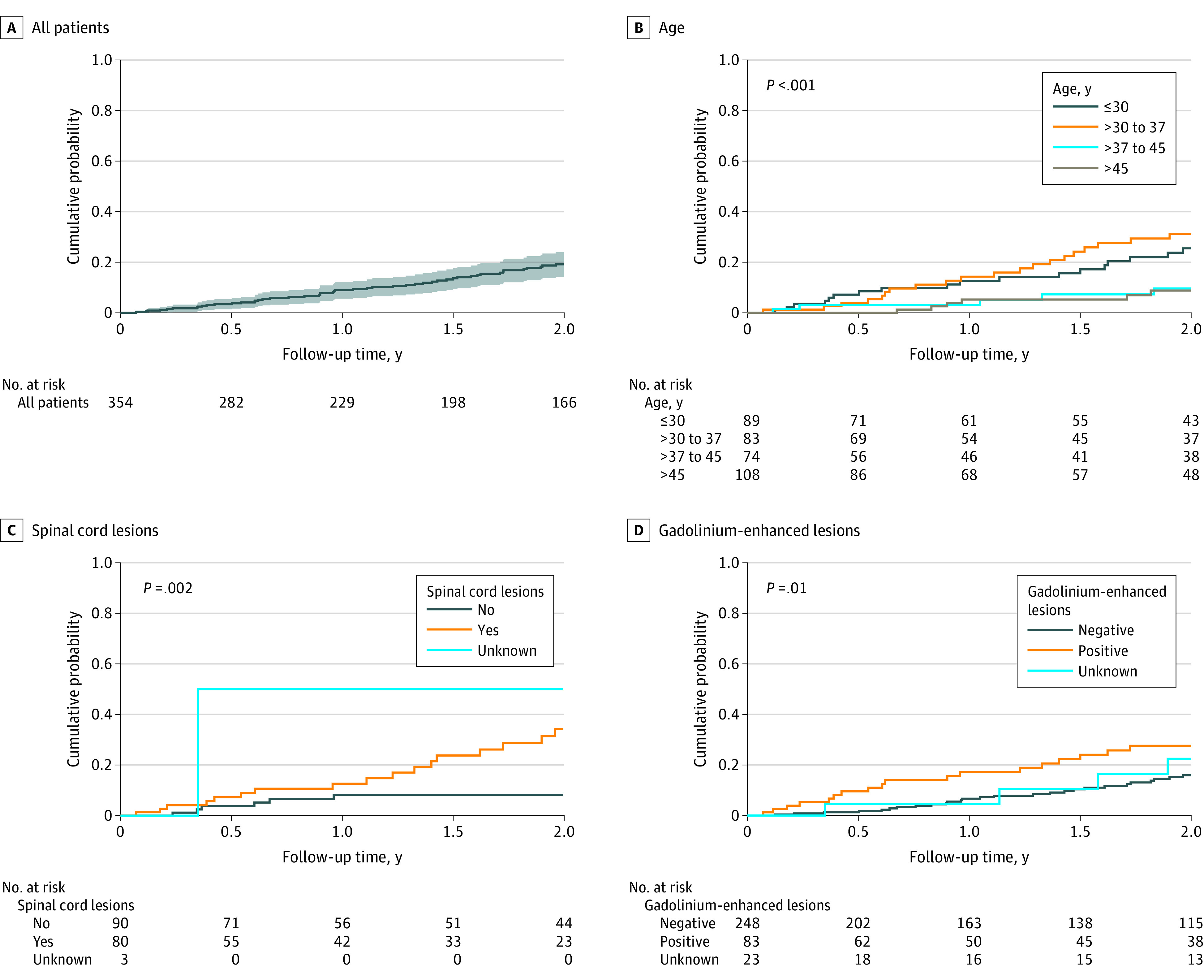
Kaplan-Meier Survival Analysis With the End Point of Time to First Acute or Progressive Event Suggestive of Multiple Sclerosis at 2 Years A, At 2 years, 49 patients (19.2% [95% CI, 14.1%-24.0%]) presented with a clinical event. Shaded area indicates 95% CIs. B, The association of age with risk of a clinical event at 2 years is presented. C, The association of the presence of spinal cord lesions at baseline with risk of a clinical event is presented. D, The association of the presence of gadolinium-enhancing brain lesions at baseline with risk of a clinical event is presented.

The estimated cumulative probability of a clinical event within 2 years was 19.2% (95% CI, 14.1%-24.0%) (Figure 1A). In univariate analysis, factors at index MRI scan associated with an increased risk of a first clinical event were age younger than 37 years (HR, 3.52 [95% CI, 1.80-6.88]; *P* < .001), spinal cord lesions (HR, 3.82 [95% CI, 1.50-9.70]; *P* = .005), and gadolinium-enhancing lesions (HR, 2.11 [95% CI, 1.16-3.83]; *P* = .01) (Table 1). The use of a DMT during follow-up without adjustment was associated with an increased risk of a first clinical event (HR, 7.49 [95% CI, 4.24-13.24]; *P* < .001). Other covariates of interest were not associated with risk of a clinical event (eFigure 1 in Supplement 1). MRI motive was not associated with a clinical event (Table 1), and T2 lesion locations were not associated with DMT use (Table 2). A statistically significantly increased percentage of patients receiving a DMT had more than 1 gadolinium lesion compared with patients without more than 1 such lesion (23 patients [37.7%] vs 60 patients [20.5%]; *P* = .004). Other demographic factors were not associated with differences in the percentage of patients receiving DMTs (Table 2).

**Table 1.** Univariate and Multivariate Analysis for First Clinical Demyelinating Event

Variable	Patient population, No.	Univariate analysis		Multivariate analysis	
		HR (95% CI)	*P* value	HR (95% CI)	*P* value
Age <37 y	354	3.52 (1.80-6.88)	<.001	4.04 (2.00-8.15)	<.001
Women	354	1.27 (0.68-2.36)	.45	NA	NA
Positive family MS history	354	0.86 (0.36-1.81)	.73	NA	NA
MRI motive (headache)	354	0.64 (0.32-1.28)	.21	NA	NA
Positive CSF IgG index >0.7l or presence of >2 unique OCBs	354	1.26 (0.51-3.09)	.61	NA	NA
Not >3 periventricular lesions	349	1.59 (0.57-4.45)	.38	NA	NA
Infratentorial lesion presence	349	1.76 (0.98-3.15)	.06	NA	NA
Juxtacortical lesion presence	349	NA^a^	NA	NA	NA
Spinal cord lesion presence	173	3.82 (1.50-9.70)	.005	5.11 (1.99-13.13)	.001
Gadolinium-enhancing lesions on index MRI	354	2.11 (1.16-3.83)	.01	2.09 (1.33-3.87)	.02
Disease modifying treatment)	61	7.49 (4.24-13.24)	<.001	0.85 (0.26-2.81)	.79

Abbreviations: CSF, cerebrospinal fluid; HR, hazard ratio; MRI, magnetic resonance imaging; MS, multiple sclerosis; NA, not applicable; OCB, oligoclonal band.

^a^ This result was not estimable given that no clinical conversion was observed for patients with juxtacortical lesions.

**Table 2.** Demographic Characteristics of Patients With RIS Receiving Treatment vs No Treatment

Characteristic	Patients with RIS, No. (%)		*P* value
	Receiving DMT (n = 61 [17.2%])	Not receiving DMT (n = 311 [82.8%])	
Sex			
Men	19 (31.2)	71 (24.2)	.26
Women	42 (68.8)	222 (75.8)	
Age, y			
<37	36 (59.0)	136 (46.4)	.07
>37	25 (41.0)	157 (53.6)	
MS family history			
Yes	10 (16.4)	36 (12.3)	.66
No	39 (63.9)	201 (68.6)	
Unknown	12 (19.7)	56 (19.1)	
CSF IgG index >0.7 or presence of >2 unique OCBs			
Positive	31 (50.8)	121 (41.3)	.24
Negative	5 (8.2)	44 (15.0)	
Unknown	25 (41.0)	128 (43.7)	
>3 periventricular T2 lesions^a^			
Yes	46 (92.0)	247 (94.6)	.46
No	4 (8.0)	14 (5.4)	
Unknown	11	32	
>1 infratentorial T2 lesion^a^			
Yes	29 (52.7)	112 (40.1)	.08
No	26 (47.3)	167 (59.9)	
Unknown	6	14	
>1 spinal cord T2 lesion (n = 173)^a^			
Yes	20 (54.1)	60 (45.1)	.33
No	17 (45.9)	73 (54.9)	
Unknown	1	2	
>1 gadolinium lesion			
Yes	23 (37.7)	60 (20.5)	.004
No	32 (52.5)	216 (73.7)	
Unknown	6 (9.8)	17 (5.8)	

Abbreviations: CSF, cerebrospinal fluid; DMT, disease-modifying therapy; MS, multiple sclerosis; OCB, oligoclonal band.

^a^ Statistical analysis was performed using yes vs no, so the analysis did not include unknown and percentages not included for unknown.

In multivariable analysis, age younger than 37 years (HR, 4.04 [95% CI, 2.00-8.15]; *P* = <.001), spinal cord lesions (HR, 5.11 [95% CI, 1.99-13.13]; *P* = .001), and gadolinium-enhancing lesions on index scan (HR, 2.09 [95% CI, 1.13-3.87]; *P* = .02) were identified as independently associated with an early clinical event (Figure 1). At 2 years, the cumulative probability for a clinical event was 53.8% (95% CI, 37.2%-66.0%) among patients receiving a DMT and 10.6% (95% CI, 6.2%-14.9%) among patients who were not treated (*P* < .001). When considering DMTs as a time-varying covariate to reduce bias, there was no statistically significant change in risk (HR, 0.85 [95% CI, 0.26-2.81]; *P* = .79).

### MRI Characteristics

Baseline spinal cord imaging was performed at each study site at the treating physician's discretion and was available for 173 patients (48.9%) (eTable 3 in Supplement 1). Among these individuals, at least 1 spinal cord lesion was observed among 80 patients (46.2%). In univariate analysis, the presence of spinal cord lesions (HR, 3.82 [95% CI, 1.50-9.70]; *P* = .005) was a risk factor associated with conversion, whereas the presence of at least 3 periventricular lesions (HR, 0.63 [95% CI, 0.23-1.77]; *P* = .38) and any infratentorial lesions (HR, 1.76 [95% CI, 0.98-3.15]; *P* = .06) were not.

Data on the presence or absence of gadolinium enhancement on index MRI scan were available for 331 patients (93.5%), and contrast enhancement was observed among 83 patients (23.4%). The presence of contrast enhancement at baseline was associated with increased risk of conversion to a first clinical event within 2 years (HR, 2.11 [95% CI, 1.16-3.83]; *P* = .01). A clinical event's cumulative probability at 2 years if there was at least a gadolinium-enhancing lesion on the index scan was 27.6% (95% CI, 15.7%-37.8%). Among 354 patients, 262 individuals had another MRI scan during 2 years of follow-up; 79 of these individuals (30.2%) had a at least 1 gadolinium-enhancing lesion, and 169 individuals (64.5%) did not have gadolinium-enhancing lesions. Among these 262 patients, 33 patients developed a first clinical demyelinating event, and 18 of these individuals (54.6%) had gadolinium-enhancing lesions on follow-up, while 229 individuals did not convert within 2 years, and 61 of these individuals (26.6%) had these lesions on follow-up. Among 181 patients with MRI during follow-up and without gadolinium-enhancing lesions on index scan, 36 individuals (19.9%) had gadolinium-enhancing lesions during follow-up, including 7 patients who developed a first clinical event (eTable 4 in Supplement 1).

Among patients with gadolinium-enhancing lesions on index scan, 13 individuals developed a first clinical event within 2 years and 10 of these individuals (76.9%) had gadolinium-enhancing lesions remaining during follow-up, while 55 individuals did not convert, among whom 28 individuals (50.9%) had these lesions remaining during follow-up (eTable 4 in Supplement 1). Using a purely descriptive approach, these figures suggest that the presence of gadolinium-enhancing lesions is a factor associated with developing a clinical event at baseline and during follow-up. Multivariate analysis found that the presence of spinal cord lesions (HR, 5.11 [95% CI, 1.99-13.13]; *P* = .001) and contrast enhancement on index MRI scan (HR, 2.09 [95% CI, 1.33-3.87]; *P* = .02) were associated with the occurrence of a clinical event at 2 years.

### Risk Stratification

**Figure 2.**
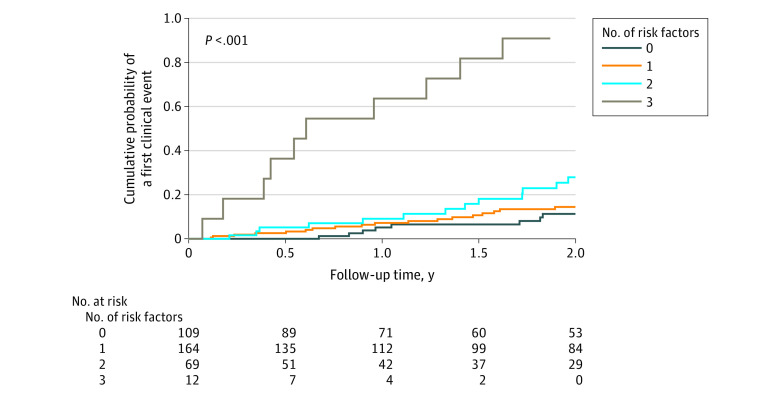
Stratification for a Clinical Event Suggestive of Multiple Sclerosis by Number of Risk Factors Among patients with 3 factors, 90.9% (95% CI, 41.1%-98.6%) had a clinical event at 2 years.

Considering 3 independent risk factors associated with an early clinical event emerging from multivariable analysis (Table 1), the probability of an early conversion was 11.3% (95% CI, 3.5%-18.5%) when no risk factor was present, 14.5% (95% CI, 8.0%-20.5%) with 1 risk factor, and 27.9% (95% CI, 13.5%-39.9%) with 2 risk factors. The probability was 90.9% (95% CI, 41.1%-98.6%) for a clinical event at 2 years among individuals with 3 risk factors at baseline (3 risk factors vs none: HR, 23.34 [95% CI, 9.08%-59.96%]; *P* < .001) (Figure 2).

### Sample Size Calculations

**Figure 3.**
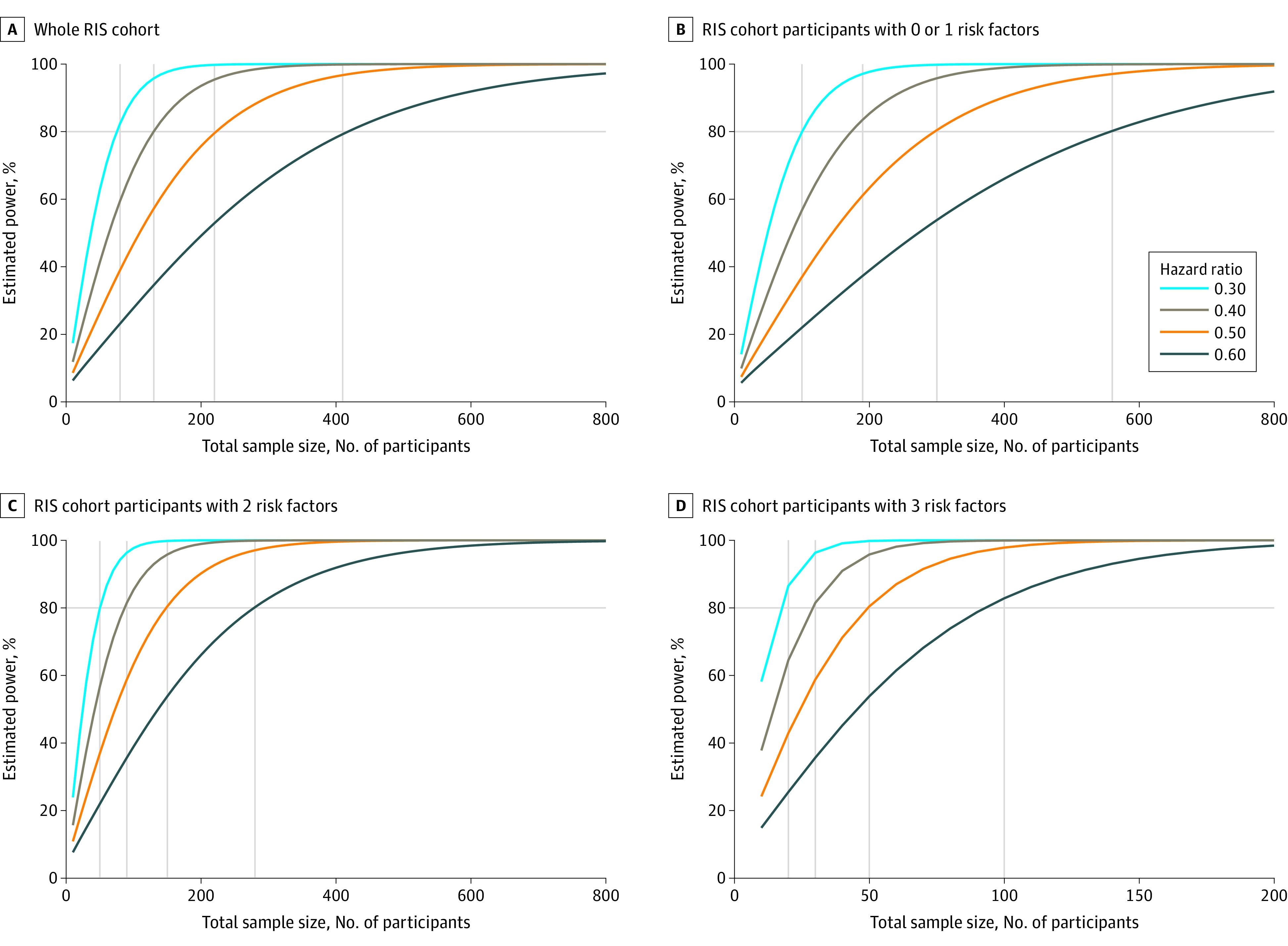
Sample Size Calculation for a 2-Year Radiologically Isolated Syndrome (RIS) Study Using 2009 RIS Criteria Overall event rates of 15%, 20%, 30%, and 90% and an SD on the covariate of 0.6 were assumed. Power function from a simulation procedure estimating total sample sizes that would be needed to detect 40% (dark blue), 50% (orange), 60% (brown), and 70% (light blue) effect sizes, assuming a 1-sided α of .05 is presented. A, Estimated power for the entire RIS cohort with 20% cumulative probability of a clinical event at 2 years is presented. B, Patients with RIS and 0 or 1 risk factors, with 15% cumulative probability, are presented. C, Patients with RIS and 2 risk factors, with 30% cumulative probability, are presented. D, Patients with RIS and 3 identified risk factors, with 90% cumulative probability, are presented.

The number of individuals with RIS needed per arm to detect a given decrease in risk for a seminal event based on the number of identified risk factors and magnitude of decreased risk within a 24-month clinical trial is shown in Figure 3. We performed sample size calculations using a 1-sided α of .05%, beginning with a 20% event rate at 2 years (with SD, 0.6). The obtained results were based on HR models (ie, treatment effects) ranging from 40% to 70%. For example, assuming a 60% treatment effect size (HR, 0.40), a total of 160 individuals with RIS and 2 years of follow-up would be needed to obtain 80% power to detect a decreased in the risk of a clinical event in a 2-arm clinical trial (Figure 3). Suppose the estimation was enriched for risk factors. In that case, the total sample size changed as follows: the number of patients needed for 1 risk factor was 200 individuals assuming an event rate of 15%, 90 individuals for 2 risk factors assuming an event rate of 30%, and 30 individuals for 3 risk factors assuming an event rate of 90%. Multiple scenarios can be visualized from the figure.

## Discussion

In this large prospective cohort study of individuals with RIS from French tertiary MS centers, we investigated the association of demographic characteristics, MRI features, and paraclinical markers with disease evolution, as measured by the occurrence of a first clinical event consistent with CNS demyelination at 2 years. Age younger than 37 years and the presence of spinal cord lesions and gadolinium-enhancing lesions at RIS diagnosis were associated with increased risk of a seminal clinical event, findings similar to those of our previous retrospective studies.^11,12,13^ Given that the field is transitioning the focus of study to earlier demyelinating disease detection, the determination of sample size estimates was performed such that these data may be applied in the development of efficient clinical trial designs aimed at studying the impact of immunomodulatory or immunosuppressant therapy in the prevention of a clinical event. We found that in the RISC and OFSEP cohorts, patients with RIS had a mean age of approximately 39 years, 8 years older than the mean age of MS onset in previous exhaustive published cohorts.^16,22,23^ An earlier study^24^ found that individuals with RIS were older than patients with clinically isolated syndrome (CIS) at diagnosis and developed their first clinical event at an older age. They also had a slower rate of developing a second clinical event compared with individuals who presented with CIS. One explanation may be that they have compensatory mechanisms associated with a silent disease.^24^

RIS was defined more than 10 years ago, with diagnostic criteria validated within a worldwide cohort.^3,11^ The search for practical and reliable clinical and radiological markers associated with risk for disease progression and, more precisely, the occurrence of clinical symptoms has been ongoing for more than a decade. In the most extended available follow-up of individuals with RIS, younger age, the presence of oligoclonal bands on the CSF profile, and the presence of infratentorial or spinal cord lesions were associated with increased risk, with an estimated 10-year risk of 51% for an acute or progressive demyelinating event.^13^ Gadolinium-enhancing lesions on follow-up scans were associated with the occurrence of a first clinical symptom, suggesting the importance of routine MRI surveillance. Although these identified risk factors appeared biologically plausible and somewhat consistent with risks in symptomatic groups, confirmation within a prospective RIS cohort was not yet available until now.

An association between the reasons for undergoing MRI and the outcome of a seminal event was also explored. In our cohort, 101 individuals (28.5%) had index MRI scans to investigate headaches. Although an association between deep periaqueductal gray matter and superficial cortical involvement with headache may exist with frequencies observed in MS cohorts ranging from 1.6% to 28.5%, we did not identify this symptom as a predictive factor associated with a clinical event within 2 years.^22,25^

Two multicenter, randomized, double-blinded clinical trials are ongoing, studying the impact of approved MS treatments in delaying the time to MS diagnosis. Based on our sample size estimates in this study and the infrequent identification of individuals who fulfill 2009 RIS criteria, the number of included patients may be scaled based on the number of selected risk factors. However, attempts to enrich a RIS cohort for clinical events may impose strict inclusion criteria. Therefore, it may not be generalizable to commonly encountered experiences in routine practice, which may be associated with in delays in effective study recruitment.

Although some informative clinical markers of disease course are available, the individual prognosis in RIS remains uncertain, leading to difficulties in counseling newly diagnosed patients. We have found that spinal cord, infratentorial, and gadolinium-enhancing lesions on index scan are associated with the occurrence of a clinical event. A 2019 study^23^ on a large CIS cohort aimed to determine which early MRI features best predict future physical and cognitive disability, including conversion to secondary progressive MS at 15 years. Early MRI lesions were positively associated with EDSS score at 15 years, including gadolinium-enhancing and spinal cord lesions at baseline and enhancing lesions at 1 and 3 years. Given that the search for novel biomarkers associated with future disease activity remains highly active, readily available data appear meaningful in predicting outcomes at an early phase of demyelinating disease.

### Strengths and Limitations

One major strength of our study is its prospective structure. To our knowledge, it provides the first evidence of risk from a large cohort of patients with RIS obtained through MS tertiary centers. Consistent medical follow-up was accomplished, with a low number of patients lost to follow-up. We used standardized definitions and a uniform MRI protocol with dual reading centers to apply the validated 2009 RIS criteria.

This study also has several limitations. Data reported here were limited to a 2-year period. Nevertheless, we found risk factors previously identified in retrospective studies and performed sample size calculations for potential future 24-month phase III trials. Additionally, not all patients underwent baseline spinal cord MRI scans and CSF analyses owing to differences in regional practice patterns. To avoid selection bias, we did not exclude patients who did not have CSF or spinal cord MRI data available, but this may explain why we did not find that positive CSF was associated with the early occurrence of MS. Our findings may also not be generalizable to the experience of other centers with patient populations with varying races and ethnicities.

## Conclusions

In this first prospective study of individuals with RIS, to our knowledge, we found that MS occurred in more than 90% of individuals within 2 years of index scan among young patients with spinal cord and gadolinium-enhancing lesions. With the widespread use of MRI technology and expansion of access throughout the world, an increase in the recognition of individuals with abnormal MRI results suggesting incidental nonspecific white matter findings is anticipated, along with an increase in the recognition of those with results highly supportive of CNS demyelination. The timing of the release of our data may be essential in guiding future study designs. The expansion of therapeutic trials in RIS in the upcoming years is needed given that the disease's earlier recognition is targeted. Our findings also suggest the need for worldwide collaboration to advance knowledge in the preclinical space's early mechanisms. New strategies for effective management and surveillance may be developed.

Our findings are time relevant given the new era of MS focused on understanding not only the very early preclinical mechanisms associated with demyelinating injury along with prodromal experiences,^26^ but also the prevention of clinical manifestations with subsequent freedom from risk of neurological disability. Historically, the focus was centered on evaluating risk factors associated with the conversion from CIS to MS. Given recent scientific advancements, RIS may be well-positioned to serve as a new focal point in our scientific efforts to more accurately and at an earlier stage recognize, guide, and possibly treat to optimize care.

## Notes

Supplement 1. **eTable 1.** Participating Centers and Investigators **eTable 2.** Disease-Modifying Treatments Prescribed to Patients with Radiologically Isolated Syndrome **eTable 3.** Magnetic Resonance Imaging Scan Characteristics **eTable 4.** Association of Gadolinium-Enhancing Lesions at Index Scan or Follow-up With Clinical Event Outcome **eFigure 1.** Demographic Factors Not Associated With an Early Clinical Event

Supplement 2. **Nonauthor Collaborators.** RISC, SFSEP, and OFSEP Investigators
