# Supplementary material for: Risk Factors and Time to Clinical Symptoms of Multiple Sclerosis Among Patients With Radiologically Isolated Syndrome
Source: JAMA Netw Open. 2021 Oct 11;4(10):e2128271. doi: 10.1001/jamanetworkopen.2021.28271 (PMC8506228; doi:10.1001/jamanetworkopen.2021.28271)
Supplement: Supplement 1. — eTable 1. Participating Centers and Investigators eTable 2. Disease-Modifying Treatments Prescribed to Patients with Radiologically Isolated Syndrome eTable 3. Magnetic Resonance Imaging Scan Characteristics eTable 4. Association of Gadolinium-Enhancing Lesions at Index Scan or Follow-up With Clinical Event Outcome eFigure 1. Demographic Factors Not Associated With an Early Clinical Event [file jamanetwopen-e2128271-s001.pdf]

Supplemental Online Content

Lebrun-Frénay C, Rollot F, Mondot L, et al; RISC, SFSEP, and OFSEP Investigators. Risk factors and time to clinical symptoms of multiple sclerosis among patients with radiologically isolated syndrome. *JAMA Netw Open*. 2021;4(10):e2128271. doi:10.1001/jamanetworkopen.2021.28271

eTable 1. Participating Centers and Investigators
eTable 2. Disease-Modifying Treatments Prescribed to Patients with Radiologically Isolated Syndrome
eTable 3. Magnetic Resonance Imaging Scan Characteristics
eTable 4. Association of Gadolinium-Enhancing Lesions at Index Scan or Follow-up With Clinical Event Outcome
eFigure 1. Demographic Factors Not Associated With an Early Clinical Event

This supplemental material has been provided by the authors to give readers additional information about their work.

© 2021 Lebrun-Frénay C et al. *JAMA Network Open.*

eTable 1. Participating Centers and Investigators

MS Centers	N= 372	%	Investigators
**Nice**	75	20.2	C Lebrun-Frénay<br>M Cohen<br>L Mondot<br>C Callier
**Lille**	41	11	H Zephir<br>P Vermersch
**Montpellier**	41	11	P Labauge<br>C Carra Dallière
**Paris Salpetrière**	33	8.9	C Louapre
**Rennes**	24	6.4	E Le Page
**Lyon**	20	5.4	F Durand-Dubief<br>F Rollot (statistics)<br>S Vukusic
**Paris F Rothschild**	18	4.8	C Bensa
**Nîmes**	16	4.3	E Thouvenot
**Toulouse**	16	4.3	J Ciron
**Bordeaux**	15	4	B Brochet
**Nancy**	14	3.8	G Mathey
**Besançon**	10	2.7	E Berger
**Dijon**	7	1.9	T Moreau
**Nantes**	6	1.6	DA Laplaud
**Strasbourg**	6	1.6	J de Seze
**Amiens**	5	1.3	A Alkhedr
**Caen**	5	1.3	N Derache
**Poitiers**	4	1.1	JP Neau
**Rouen**	4	1.1	B Bourre
**Grenoble**	3	0.8	O Casez
**Clermont-Ferrand**	2	0.5	P Clavelou
**Fort de France**	2	0.5	P Cabre
**Tours**	2	0.5	AM Guennoc
**Corbeil**	1	0.3	C Dussaule
**Creteil**	1	0.3	A Créange
**Saint-Etienne**	1	0.3	JP Camdessanché

© 2021 Lebrun-Frénay C et al. *JAMA Network Open.*

eTable 2. Disease-Modifying Treatments Prescribed to Patients with Radiologically Isolated Syndrome

Drug	Number	%
**Interferon-beta 1A**	13	17.3
**Teriflunomide**	13	17.3
**Dimethyl Fumarate**	9	12.0
**Glatiramer Acetate**	7	9.3
**Interféron beta 1B**	5	6.7
**Natalizumab**	5	6.7
**Fingolimod**	4	5.3
**Azathioprine**	1	1.3
**Methotrexate**	1	1.3
**Mitoxantrone**	1	1.3
**Mycophenolate Mofetil**	1	1.3
**Peginterferon beta 1A**	1	1.3
**Rituximab**	1	1.3
**Clinical study**	13	17.3
**Total**	**75**	

© 2021 Lebrun-Frénay C et al. *JAMA Network Open.*

eTable 3. Magnetic Resonance Imaging Scan Characteristics

**Only 1 periventricular lesion**	N	%
**Yes**	6	**1.6**
**no**	323	86.8
**Unknown**	38	10.2
**>3 periventricular lesions**		
**Yes**	310	**83.3**
**no**	19	5.1
**Unknown**	38	10.2
**>1 Juxtacortical lesion**		
**Yes**	291	**78.2**
**no**	19	5.1
**Unknown**	38	10.2
**>1 infratentorial lesion**		
**Yes**	57	**40.6**
**no**	201	54.0
**Unknown**	15	4
**>1 Spinal Cord lesion**		
**Yes**	82	22.0
**no**	92	24.7
**Unknown**	15	0.8
**No spinal cord MRI**	195	**52.4**
**>1 Gadolinium lesion**		
**Yes**	88	**23.7**
**no**	261	70.2
**Unknown**	23	6.2

© 2021 Lebrun-Frénay C et al. *JAMA Network Open.*

eTable 4. Association of Gadolinium-Enhancing Lesions at Index Scan or Follow-up With Clinical Event Outcome

	Clinical event within 2 years		
	**No**	**Yes**	**Total**
	**N (%)**	**N (%)**	**N (%)**
**Gadolinium-enhancing lesions during follow-up[1]**			
Presence	61 (26.6)	18 (54.6)	79 (30.2)
Absence	155 (67.7)	14 (42.4)	169 (64.5)
Unknown	13 (5.7)	1(3.0)	14 (5.3)
For patients with a presence of gadolinium-enhancing lesions at index scan[2]			
**Gadolinium-enhancing lesions during follow-up**			
Presence	28 (50.9)	10 (76.9)	38 (55.9)
Absence	25 (45.4)	3 (23.1)	28 (41.2)
Unknown	2 (3.6)	0 (0)	2 (2.9)
For patients with an absence of gadolinium-enhancing lesions at index scan[3]			
**Gadolinium-enhancing lesions during follow-up**			
Presence	29 (17.8)	7 (38.9)	36 (19.9)
Absence	125 (76.7)	10 (55.6)	135 (74.6)
Unknown	9 (5.5)	1 (5.6)	10 (5.5)

[1] 92 patients without MRI during follow-up
[2] 15 patients without MRI during follow-up
[3] 67 patients without MRI during follow-up

eFigure 1. Demographic Factors Not Associated With an Early Clinical Event

A. Sex. B. CSF positivity. C. Familial history of MS. D. Headache as MRI motive

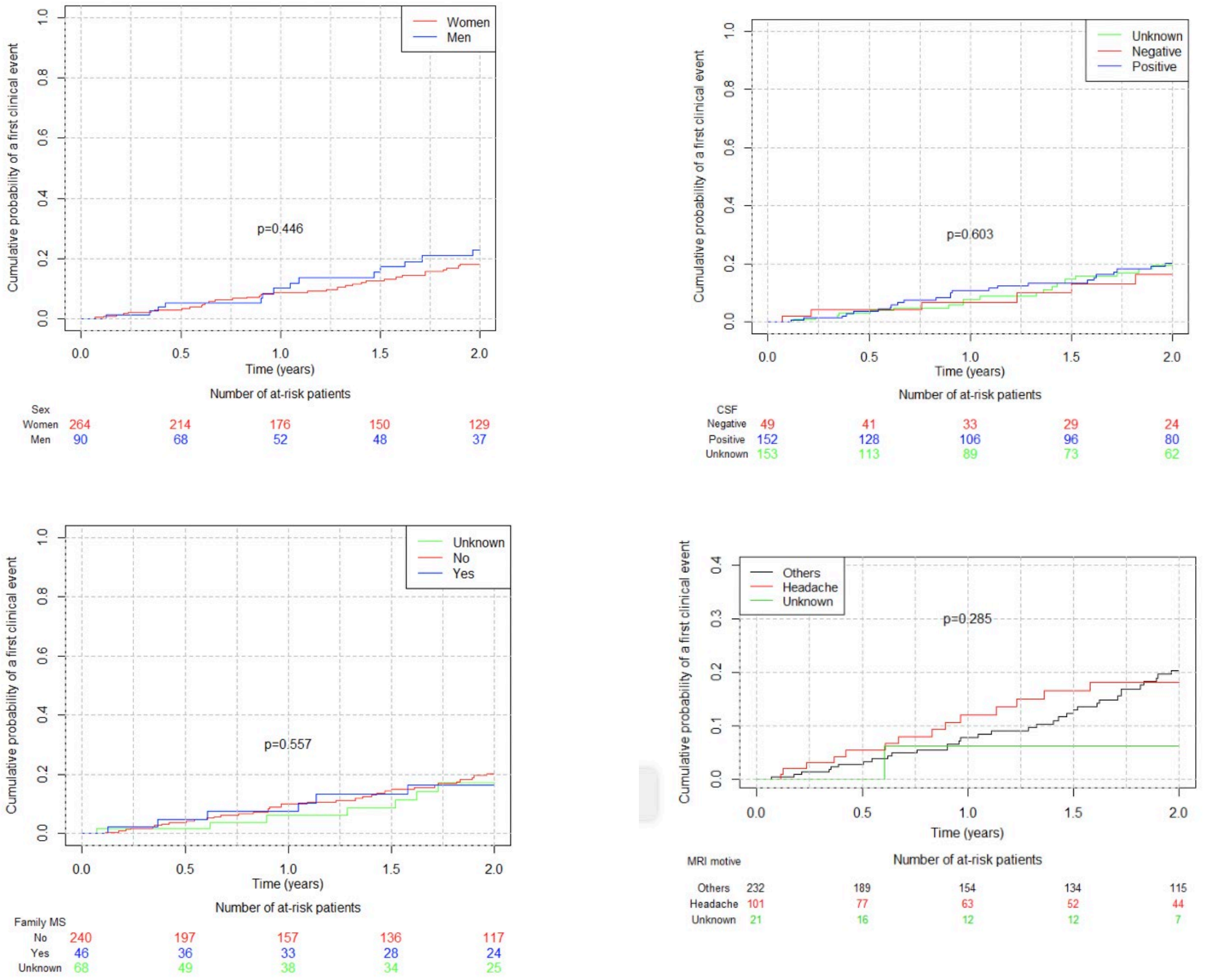

© 2021 Lebrun-Frénay C et al. *JAMA Network Open.*
