# Supplementary material for: Risk Factors and Time to Clinical Symptoms of Multiple Sclerosis Among Patients With Radiologically Isolated Syndrome
Source: JAMA Netw Open. 2021 Oct 11;4(10):e2128271. doi: 10.1001/jamanetworkopen.2021.28271 (PMC8506228; doi:10.1001/jamanetworkopen.2021.28271)
Supplement: Supplement 2. — Nonauthor Collaborators. RISC, SFSEP, and OFSEP Investigators [file jamanetwopen-e2128271-s002.pdf]

Group Name(s): RISC Investigators, SFSEP Investigators, OFSEP Investigators

First Name and Middle Initial(s)	Last Name	Suffix (eg, Jr, III)	Academic Degrees	Institution	Location (city, state/province, country)	Role or Contribution, eg, chair, principal investigator	Group (if more than 1 Group listed in the byline) and/or Subgroup (eg, Steering Committee)
Orhun	Kantarci		MD	*Mayo Clinic*	*Rochester, MN, USA*	DATA PROVIDER	RISC Investigators, OFSEP Steering Committee
Aksel	Siva		MD	*Istanbul University Cerrahpa*	*Istanbul,Turkey*	DATA PROVIDER	RISC Investigators, OFSEP Steering Committee
Daniel	Pelletier		MD	*University of Southern Califo*	*Los Angeles, CA, USA*	DATA PROVIDER	RISC Investigators, OFSEP Steering Committee
Darin	Okuda		MD	*University of Texas SouthWe*	*Dallas, TX, USA*	DATA PROVIDER, RISC Coordinator	RISC Investigators, OFSEP Steering Committee
Christina	Azevedo		MD	*University of Southern Califo*	*Los Angeles, CA, USA*	DATA PROVIDER	RISC Investigators, Steering Committee
Naila	Makhani		MD	*Departments of Pediatrics ar*	*New Haven, CT, USA*	DATA PROVIDER, Pediatric RISC Coordinator	RISC Investigators, Steering Committee
Fatai	Radji		MD	*Neurologist*	*Agens, France*	DATA PROVIDER	SFSEP Investigators France
Nathalie	Morel		MD	*Neurologist*	*Annecy, France*	DATA PROVIDER	SFSEP Investigators France
Deborah	Grosset-Jeannin		MD	*Neurologist*	*Annecy, France*	DATA PROVIDER	SFSEP Investigators France
Aurelian	Ungureanu		MD	*Neurologist*	*Annecy, France*	DATA PROVIDER	SFSEP Investigators France
Latine	Boyer		MD	*Neurologist*	*La Rochelle, France*	DATA PROVIDER	SFSEP Investigators France
Laurent	Suchet		MD	*Neurologist*	*Marseille, France*	DATA PROVIDER	SFSEP Investigators France
Zyad	Elias		MD	*Neurologist*	*Toulon, France*	DATA PROVIDER	SFSEP Investigators France
Christine	Lebrun-Frénay		MD, PhI	*Nice University Hospital, UF*	*Nice, France*	DATA PROVIDER, OFSEP scientific committee Co-president, RISC Coordinator	OFSEP Investigators France, SFSEP Investigator, RISC investigator
Mikael	Cohen		MD	*Nice University Hospital, UF*	*Nice, France*	DATA PROVIDER, RISC Steering committee	OFSEP Investigators France, SFSEP Investigator, RISC investigator France
Lydiane	Mondot		MD	*Nice University Hospital, UF*	*Nice, France*	DATA PROVIDER, RISC Steering committee	OFSEP Investigators France, SFSEP Investigator, RISC investigator France
Eric	Thouvenot		MD, PhI	*Nîmes University Hospital*	*Nîmes, France*	DATA PROVIDER, OFSEP Operational scientific committee, SFSEP President	OFSEP Investigators France, SFSEP Investigator, RISC investigator France
Jonathan	Ciron		MD	*Toulouse University Hospita*	*Toulouse, France*	DATA PROVIDER, RISC Steering committee	OFSEP Investigators France, SFSEP Investigator France, RISC Investigator France
Françoise	Durand-Dubief		MD, PhI	*Hospices Civils de Lyon*	*Lyon, France*	DATA PROVIDER, OFSEP operational Scientific committee, RISC Steering committee	OFSEP Investigators France, SFSEP Investigator, RISC investigator France
Bruno	Brochet		MD, PhI	*Bordeaux University Hospita*	*Bordeaux, France*	DATA PROVIDER, OFSEP Operational committee	OFSEP Investigators France, SFSEP Investigator France

First Name and Middle Initial(s)	Last Name	Suffix (eg, Jr, III)	Academic Degrees	Institution	Location (city, state/province, country)	Role or Contribution, eg, chair, principal investigator	Group (if more than 1 Group listed in the byline) and/or Subgroup (eg, Steering Committee)
Aurélie	Ruet		MD, PhI	*Bordeaux University Hospita*	*Bordeaux, France*	DATA PROVIDER	OFSEP Investigators France, SFSEP Investigator France
Jean-Christophe	Ouallet		MD	*Bordeaux University Hospita*	*Bordeaux, France*	DATA PROVIDER	OFSEP Investigators France, SFSEP Investigator France
Gilles	Defer		MD, PhI	*Caen University Hospital*	*Caen, France*	DATA PROVIDER	OFSEP Investigators France, SFSEP Investigator France
Pierre	Branger		MD	*Caen University Hospital*	*Caen, France*	DATA PROVIDER	OFSEP Investigators France, SFSEP Investigator France
Nathalie	Derache		MD	*Caen University Hospital*	*Caen, France*	DATA PROVIDER	OFSEP Investigators France, SFSEP Investigator France
Pierre	Clavelou		MD, PhI	*Clermont-Ferrand University*	*Clermont-Ferrand, Fr*	DATA PROVIDER	OFSEP Investigators France, SFSEP Investigator France
Frédéric	Taithe		MD	*Clermont-Ferrand University*	*Clermont-Ferrand, Fr*	DATA PROVIDER	OFSEP Investigators France, SFSEP Investigator France
Adullatif	Alkhedr		MD	*Amiens University Hospital*	*Amiens, France*	DATA PROVIDER	OFSEP Investigators France, SFSEP Investigator France
Agnès	Fromont		MD, PhI	*Dijon University Hospital*	*Dijon, France*	DATA PROVIDER	OFSEP Investigators France, SFSEP Investigator France
Thibault	Moreau		MD, PhI	*Dijon University Hospital*	*Dijon, France*	DATA PROVIDER, OFSEP Operational committee	OFSEP Investigators France, SFSEP Investigator France
Ayman	Tourbah		MD, PhI	*APHP IdF Ouest, Paris Sacl*	*Paris, France*	DATA PROVIDER	OFSEP Investigators France, SFSEP Investigator France
Olivier	Casez		MD	*Grenoble-Alpes University H*	*Grenoble, France*	DATA PROVIDER	OFSEP Investigators France, SFSEP Investigator France
Eric	Berger		MD	*Besancon University Hospita*	*Besancon, France*	DATA PROVIDER	OFSEP Investigators France, SFSEP Investigator France
Helene	Zephir		MD, PhI	*Lille University Hospital*	*Lille, France*	DATA PROVIDER, OFSEP scientific committee Co-president	OFSEP Investigators France, SFSEP Investigator France
Olivier	Outteryck		MD, PhI	*Lille University Hospital*	*Lille, France*	DATA PROVIDER	OFSEP Investigators France, SFSEP Investigator France
Patrick	Vermersch		MD, PhI	*Lille University Hospital*	*Lille, France*	DATA PROVIDER	OFSEP Investigators France, SFSEP Investigator France
Patrick	Hautecoeur		MD, PhI	*Lille Catholic University*	*Lille, France*	DATA PROVIDER	OFSEP Investigators France, SFSEP Investigator France
Geraldine	Androdias		MD	*Hospices Civils de Lyon*	*Lyon, France*	DATA PROVIDER	OFSEP Investigators France, SFSEP Investigator France
Iuliana	Ionescu		MD	*Hospices Civils de Lyon*	*Lyon, France*	DATA PROVIDER	OFSEP Investigators France, SFSEP Investigator France
Jean	Pelletier		MD, PhI	*Assistance Publique des Hôp*	*Marseille, France*	DATA PROVIDER, OFSEP Operational committee	OFSEP Investigators France, SFSEP Investigator France
Bertrand	Audoin		MD, PhI	*Assistance Publique des Hôp*	*Marseille, France*	DATA PROVIDER	OFSEP Investigators France, SFSEP Investigator France
Adil	Maarouf		MD, PhI	*Assistance Publique des Hôp*	*Marseille, France*	DATA PROVIDER	OFSEP Investigators France, SFSEP Investigator France
Xavier	Ayrignac		MD, PhI	*Montpellier University Hosp*	*Montpellier, France*	DATA PROVIDER	OFSEP Investigators France, SFSEP Investigator France
Clarisse	Carra-Dalliere		MD	*Montpellier University Hosp*	*Montpellier, France*	DATA PROVIDER	OFSEP Investigators France, SFSEP Investigator France
Pierre	Labauge		MD, PhI	*Montpellier University Hosp*	*Montpellier, France*	DATA PROVIDER	OFSEP Investigators France, SFSEP Investigator France
Marc	Debouverie		MD, PhI	*Nancy University Hospital*	*Nancy, France*	DATA PROVIDER	OFSEP Investigators France, SFSEP Investigator France
Guillaume	Mathey		MD	*Nancy University Hospital*	*Nancy , France*	DATA PROVIDER	OFSEP Investigators France, SFSEP Investigator France
Philippe	Cabre		MD, PhI	*Martinique University Hospi*	*Fort de France, Fran*	DATA PROVIDER	OFSEP Investigators France, SFSEP Investigator France
David	Laplaud		MD, PhI	*Nantes University Hospital*	*Nantes, France*	DATA PROVIDER, OFSEP Operational committee	OFSEP Investigators France, SFSEP Investigator France
Sandrine	Wiertlevski		MD	*Nantes University Hospital*	*Nantes, France*	DATA PROVIDER	OFSEP Investigators France, SFSEP Investigator France
Saskia	Bresch		MD	*Nice University Hospital, UR*	*Nice, France*	DATA PROVIDER	OFSEP Investigators France, SFSEP Investigator France

**First Name and Middle Initial(s)**	**Last Name**	**Suffix (eg, Jr, III)**	Academic Degrees	Institution	Location (city, state/province, country)	Role or Contribution, eg, chair, principal investigator	Group (if more than 1 Group listed in the byline) and/or Subgroup (eg, Steering Committee)
Giovanni	Castelnovo		MD	*Nîmes University Hospital*	*Nîmes, France*	DATA PROVIDER	OFSEP Investigators France, SFSEP Investigator France
Caroline	Papeix		MD	*Assistance Publique des Hôp*	*Paris, France*	DATA PROVIDER	OFSEP Investigators France, SFSEP Investigator France
Celine	Louapre		MD, PhI	*Assistance Publique des Hôp*	*Paris, France*	DATA PROVIDER	OFSEP Investigators France, SFSEP Investigator France
Elisabeth	Maillart		MD	*Assistance Publique des Hôp*	*Paris, France*	DATA PROVIDER, OFSEP Operational scientific committee	OFSEP Investigators France, SFSEP Investigator France
Catherine	Lubetzki		MD, PhI	*Assistance Publique des Hôp*	*Paris, France*	DATA PROVIDER	OFSEP Investigators France, SFSEP Investigator France
Bruno	Stankoff		MD, PhI	*Assistance Publique des Hôp*	*Paris, France*	DATA PROVIDER, OFSEP Operational committee	OFSEP Investigators France, SFSEP Investigator France
Bertrand	Fontaine		MD, PhI	*Sorbonne University*	*Paris, France*	DATA PROVIDER	OFSEP Investigators France, SFSEP Investigator France
Claire	Giannesini		MD	*Assistance Publique des Hôp*	*Paris, France*	DATA PROVIDER	OFSEP Investigators France, SFSEP Investigator France
Olivier	Heinzleff		MD	*Poissy-Saint-Germain Hospi*	*Poissy, France*	DATA PROVIDER	OFSEP Investigators France, SFSEP Investigator France
Alain	Créange		MD, PhI	*Assistance Publique des Hôp*	*Paris, France*	DATA PROVIDER	OFSEP Investigators France, SFSEP Investigator France
Bertrand	Bourre		MD, PhI	*Rouen University Hospital*	*Rouen, France*	DATA PROVIDER	OFSEP Investigators France, SFSEP Investigator France
Olivier	Gout		MD, PhI	*Fondation Rothschild*	*Paris, France*	DATA PROVIDER	OFSEP Investigators France, SFSEP Investigator France
Antoine	Guegen		MD	*Fondation Rothschild*	*Paris, France*	DATA PROVIDER	OFSEP Investigators France, SFSEP Investigator France
Caroline	Bensa		MD	*Fondation Rothschild*	*Paris, France*	DATA PROVIDER	OFSEP Investigators France, SFSEP Investigator France
Emmanuelle	Le Page		MD	*Rennes University Hospital*	*Rennes, France*	DATA PROVIDER	OFSEP Investigators France, SFSEP Investigator France
Laure	Michel		MD, PhI	*Rennes University Hospital*	*Rennes, France*	DATA PROVIDER, OFSEP Operational committee	OFSEP Investigators France, SFSEP Investigator France
Laurent	Magy		MD, PhI	*Limoges University Hospital*	*Limoges, France*	DATA PROVIDER	OFSEP Investigators France, SFSEP Investigator France
Jerome	De Seze		MD, PhI	*Strasbourg University Hospi*	*Strasbourg, France*	DATA PROVIDER, OFSEP Operational committee	OFSEP Investigators France, SFSEP Investigator France
Nicolas	Collongues		MD, PhI	*Strasbourg University Hospi*	*Strasbourg, France*	DATA PROVIDER	OFSEP Investigators France, SFSEP Investigator France
Emmanuelle	Leray		PhD	*Ecole des Hautes Etudes en S*	*Rennes, France*	DATA PROVIDER, OFSEP Operational committee	OFSEP Investigators France, SFSEP Investigator France
Anne Marie	Guennoc		CRA	*Toulouse University Hospita*	*Tours, France*	DATA PROVIDER	OFSEP Investigators France, SFSEP Investigator France
Damien	Biotti		MD	*Toulouse University Hospita*	*Toulouse, France*	DATA PROVIDER	OFSEP Investigators France, SFSEP Investigator France
Jean-Philippe	Camdessanché		MD, PhI	*Saint-Etienne University Hos*	*Saint-Etienne, France*	DATA PROVIDER	OFSEP Investigators France, SFSEP Investigator France
Thomas	De Broucker		MD	*Saint-Denis Hospital*	*Saint-Denis, France*	DATA PROVIDER	OFSEP Investigators France
Sabrina	Sehaki		CRA	*Amiens University Hospital*	*Amiens, France*	DATA PROVIDER	OFSEP Investigators France
Nathalie	Devys-Meyer		MD	*Besancon University Hospita*	*Besancon, France*	DATA PROVIDER	OFSEP Investigators France
Mathieu	Bereau		MD	*Besancon University Hospita*	*Besancon, France*	DATA PROVIDER	OFSEP Investigators France
Chrystelle	Cappe		CRA	*Besancon University Hospita*	*Besancon, France*	DATA PROVIDER	OFSEP Investigators France
Katy-Kim	Kounkou		CRA	*Bordeaux University Hospita*	*Bordeaux, France*	DATA PROVIDER	OFSEP Investigators France
Emilie	Dumont		CRA	*Clermont-Ferrand University*	*Clermont-Ferrand, Fr*	DATA PROVIDER	OFSEP Investigators France
Edwige	Lescieux		CRA	*Corbeil-Essones Hospital*	*Corbeil-Essones, Fran*	DATA PROVIDER	OFSEP Investigators France

First Name and Middle Initial(s)	Last Name	Suffix (eg, Jr, III)	Academic Degrees	Institution	Location (city, state/province, country)	Role or Contribution, eg, chair, principal investigator	Group (if more than 1 Group listed in the byline) and/or Subgroup (eg, Steering Committee)
Alexia	Protin		CRA	*Dijon University Hospital*	*Dijon, France*	DATA PROVIDER	OFSEP Investigators France
Maty Diop	Kane		CRA	*Grenoble-Alpes University H*	*Grenoble, France*	DATA PROVIDER	OFSEP Investigators France
Julie	Boucher		CRA	*Lille University Hospital*	*Lille, France*	DATA PROVIDER	OFSEP Investigators France
Julie	Petit		CRA	*Lille University Hospital*	*Lille, France*	DATA PROVIDER	OFSEP Investigators France
Irène	Tabellah Kasonde		MD	*Limoges University Hospital*	*Limoges, France*	DATA PROVIDER	OFSEP Investigators France
Aymeric	De Vilmarrest		MD	*Limoges University Hospital*	*Limoges, France*	DATA PROVIDER	OFSEP Investigators France
Marie	Nicol		CRA	*Limoges University Hospital*	*Limoges, France*	DATA PROVIDER	OFSEP Investigators France
Muriel	Malbezin		MD	*Claude Bernard Lyon 1 Univ*	*Lyon, France*	DATA PROVIDER	OFSEP Investigators France
Javier	Olaiz		CRA	*Claude Bernard Lyon 1 Univ*	*Lyon, France*	DATA PROVIDER	OFSEP Investigators France
Claire	Rigaud-Bully		CRA	*EDMUS Foundation for Mul*	*Lyon, France*	DATA PROVIDER, OFSEP Operational committee	OFSEP Investigators France
Romain	Casey		PhD	*EDMUS Foundation for Mul*	*Lyon, France*	DATA PROVIDER, OFSEP Operational committee	OFSEP Investigators France
Fabien	Rollot		PhD	*EDMUS Foundation for Mul*	*Lyon, France*	DATA PROVIDER, OFSEP Operational committee	OFSEP Investigators France
Irena	Vukusic		RN	*EDMUS Foundation for Mul*	*Lyon, France*	DATA PROVIDER, OFSEP Operational committee	OFSEP Investigators France
Nadine	Debard		CRA	*Observatoire Français de la*	*Lyon, France*	DATA PROVIDER	OFSEP Investigators France
François	Cotton		MD, PhI	*Hospices Civils de Lyon*	*Lyon, France*	DATA PROVIDER, OFSEP Operational committee	OFSEP Investigators France
Amalle	Abdelalli		CRA	*Hospices Civils de Lyon*	*Lyon, France*	DATA PROVIDER	OFSEP Investigators France
Bernadette	Di Lelio		MA	*Assistance Publique des Hôp*	*Marseille, France*	DATA PROVIDER	OFSEP Investigators France
Frédéric	Pinna		CRA	*Montpellier University Hosp.*	*Montpellier, France*	DATA PROVIDER	OFSEP Investigators France
Francis	Guillemin		MD, PhI	*Nancy University Hospital*	*Nancy, France*	DATA PROVIDER, OFSEP Operational committee	OFSEP Investigators France
Amandine	Ziegler		CRA	*Nancy University Hospital*	*Nancy, France*	DATA PROVIDER	OFSEP Investigators France
Céline	Callier		CRA	*Nice University Hospital, UF*	*Nice, France*	DATA PROVIDER	OFSEP Investigators France
Karima	Zehrouni		CRA	*Assistance Publique des Hôp*	*Paris, France*	DATA PROVIDER	OFSEP Investigators France
Jérôme	Hodel		MD, PhI	*Assistance Publique des Hôp*	*Paris, France*	DATA PROVIDER	OFSEP Investigators France
Abir	Wahab		MD	*Assistance Publique des Hôp*	*Paris, France*	DATA PROVIDER	OFSEP Investigators France
Mickaël	Zedet		MD	*Assistance Publique des Hôp*	*Paris, France*	DATA PROVIDER	OFSEP Investigators France
Ombeline	Fagniez		MD	*Poissy-Saint-Germain Hospi*	*Poissy, France*	DATA PROVIDER	OFSEP Investigators France
Clémence	Laage		MD	*Poissy-Saint-Germain Hospi*	*Poissy, France*	DATA PROVIDER	OFSEP Investigators France
Corinne	Pottier		MD	*Pontoise Hospital*	*Pontoise, France*	DATA PROVIDER	OFSEP Investigators France
Iuliana	Slesari		MD	*Pontoise Hospital*	*Pontoise, France*	DATA PROVIDER	OFSEP Investigators France

**First Name and Middle Initial(s)**	**Last Name**	**Suffix (eg, Jr, III)**	Academic Degrees	Institution	Location (city, state/province, country)	Role or Contribution, eg, chair, principal investigator	Group (if more than 1 Group listed in the byline) and/or Subgroup (eg, Steering Committee)							
Mathilde	Sampaio		MD	*Pontoise Hospital*	*Pontoise, France*	DATA PROVIDER	OFSEP Investigators France							
Jean-Philippe	Neau		MD, PhI	*Poitiers University Hospital*	*Poitiers, France*	DATA PROVIDER	OFSEP Investigators France							
Emilie	Rabois		CRA	*Poitiers University Hospital*	*Poitiers, France*	DATA PROVIDER	OFSEP Investigators France							
Cédric	Castex		CRA	*Reims University Hospital*	*Reims, France*	DATA PROVIDER	OFSEP Investigators France							
Benjamin	Hebant		MD	*Rouen University Hospital*	*Rouen, France*	DATA PROVIDER	OFSEP Investigators France							
Maxime	Guillaume		MD	*Rouen University Hospital*	*Rouen, France*	DATA PROVIDER	OFSEP Investigators France							
Christine	Vimont		MD	*Rouen University Hospital*	*Rouen, France*	DATA PROVIDER	OFSEP Investigators France							
Romain	Muraz		CRA	*Rennes University Hospital*	*Rennes, France*	DATA PROVIDER	OFSEP Investigators France							
Damien	Le Port		CRA	*Rennes University Hospital*	*Rennes, France*	DATA PROVIDER	OFSEP Investigators France							
Carole	Henry		MD	*Saint-Denis Hospital*	*Saint-Denis, France*	DATA PROVIDER	OFSEP Investigators France							
Carole	Berthe		CRA	*Strasbourg University Hospi*	*Strasbourg, France*	DATA PROVIDER	OFSEP Investigators France							
Noellie	Freitas		MD	*Toulouse University Hospita*	*Toulouse, France*	DATA PROVIDER	OFSEP Investigators France							
Vincent	Visneux		CRA	*Saint-Etienne University Hos*	*Saint-Etienne, France*	DATA PROVIDER	OFSEP Investigators France							
Melanie	Forestier		MD	*Saint-Etienne University Hos*	*Saint-Etienne, France*	DATA PROVIDER	OFSEP Investigators France							
Stéphane	Beltran		MD	*Tours University Hospital*	*Tours, France*	DATA PROVIDER	OFSEP Investigators France							
Géraldine	Meunier		CRA	*Tours University Hospital*	*Tours, France*	DATA PROVIDER	OFSEP Investigators France							
Jérôme	Servan		MD	*Versailles University Hospita*	*Versailles, France*	DATA PROVIDER	OFSEP Investigators France							
Fernando	Pico		MD, PhI	*Versailles University Hospita*	*Versailles, France*	DATA PROVIDER	OFSEP Investigators France							
Virginie	Chatagner		CRA	*Versailles University Hospita*	*Versailles, France*	DATA PROVIDER	OFSEP Investigators France							
